# Whole-exome sequencing in moyamoya patients of Northern-European origin identifies gene variants involved in Nitric Oxide metabolism: A pilot study

**DOI:** 10.1016/j.bas.2023.101745

**Published:** 2023-04-22

**Authors:** Markus K.H. Wiedmann, Ingunn V. Steinsvåg, Tovy Dinh, Magnus D. Vigeland, Pål G. Larsson, Hanne Hjorthaug, Ying Sheng, Inger-Lise Mero, Kaja K. Selmer

**Affiliations:** a Department of Neurosurgery, The National Hospital, Oslo University Hospital, Oslo, Norway; b Department of Medical Genetics, Oslo University Hospital and the University of Oslo, Oslo, Norway; c Department of Research and Innovation, Division of Clinical Neuroscience, Oslo University Hospital, Oslo, Norway

**Keywords:** Moyamoya, Stroke, Nitric oxide, Gene, RNF213, Whole exome sequencing

## Abstract

**Introduction:**

Moyamoya disease (MMD) is a chronic cerebrovascular steno-occlusive disease of largely unknown etiology. Variants in the *RNF213* gene are strongly associated with MMD in East-Asia. In MMD patients of Northern-European origin, no predominant susceptibility variants have been identified so far.

**Research question:**

Are there specific candidate genes associated with MMD of Northern-European origin, including the known *RNF213* gene? Can we establish a hypothesis for MMD phenotype and associated genetic variants identified for further research?

**Material and methods:**

Adult patients of Northern-European origin, treated surgically for MMD at Oslo University Hospital between October 2018 to January 2019 were asked to participate. WES was performed, with subsequent bioinformatic analysis and variant filtering. The selected candidate genes were either previously reported in MMD or known to be involved in angiogenesis. The variant filtering was based on variant type, location, population frequency, and predicted impact on protein function.

**Results:**

Analysis of WES data revealed nine variants of interest in eight genes. Five of those encode proteins involved in nitric oxide (NO) metabolism: *NOS3*, *NR4A3, ITGAV, GRB7* and *AGXT2*. In the *AGXT2* gene, a *de novo* variant was detected, not previously described in MMD. None harboured the p.R4810K missense variant in the *RNF213* gene known to be associated with MMD in East-Asian patients*.*

**Discussion and conclusion:**

Our findings suggest a role for NO regulation pathways in Northern-European MMD and introduce *AGXT2* as a new susceptibility gene. This pilot study warrants replication in larger patient cohorts and further functional investigations.

## Highlights

• In this Norwegian pilot study, whole exome sequencing was performed and variants in eight candidate genes identified.
• Five of these genes are involved in NO regulation. The *AGXT2* gene, was identified as a *de novo* variant.
• Identification of a *de novo* variant in *AGXT2* supports the hypothesis of NO pathway dysregulation as a contributor in MMD.
• Identification of five other rare gene variants in our cases, in *NR4A3, ITGAV, GRB7,* *NOS3,* are all involved in NO metabolism.
• This pilot study proposes a role of NO metabolism in MMD that should be further assessed in larger patient cohorts.

## Abbreviations and acronyms:

ADMA: Asymmetric dimethylarginine
AGXT2: Alanine--Glyoxylate Aminotransferase 2
CADD: Combined Annotation-Dependent Depletion
CNV: Copy number variant
DIAPH1 –: Diaphanous Related Formin 1
GATK: Genome Analysis Toolkit
GRB7: Growth Factor Receptor Bound Protein 7
GUCY1A3: Guanylate cyclase soluble subunit alpha-3
IEL –: Internal elastic lamina
ITGAV: Integrin Subunit Alpha V
ITGB3 –: Integrin Subunit Beta 3
MMD –: Moyamoya disease
NO –: Nitric oxide
NOS3: Nitric Oxide Synthase 3
NR4A3: Nuclear Receptor Subfamily 4 Group A Member 3
PI3K: Phosphoinositide 3-kinase
RNF213: Ring finger protein 213
ROS: Reactive oxygen species
SDMA –: Symmetric dimethylarginine
SGC: Soluble guanylyl cyclase
PKB –: Protein kinase B
VEGF –: Vascular endothelial growth factor
VSMC –: Vascular smooth muscle cell
WES: Whole-exome sequencing

## 1 Introduction

Moyamoya disease (MMD) is a rare cerebrovascular condition characterized by progressive stenosis of the distal internal carotid artery and its proximal branches, with subsequent development of a compensatory collateral network at the base of the brain (Suzuki and Takaku, 1969). Disease incidence is higher in Japan, Korea and China than in Europe and North America (Kleinloog et al., 2012). Differences in disease susceptibility by ethnic background are conserved after emigration (Uchino et al., 2005). One study of 15 Japanese families with three or more members affected by MMD suggests an autosomal mode of inheritance with incomplete penetrance of familial MMD (Mineharu et al., 2006). This suggests a key role of genetic factors in MMD development. However, there is limited data on epidemiology and genetic predisposition in European patients with MMD, with only a few case reports describing familial cases (Birkeland and Lauritsen, 2018; Hever et al., 2015; Kraemer et al., 2012; Grangeon et al., 2019). The appearance of both sporadic and familial MMD cases suggests a complex mode of inheritance. Some genetic variants have more impact than others. In East-Asian populations, variants in the *RNF213* gene confer a strong susceptibility to develop disease (Liu et al., 2011; Kamada et al., 2011). In particular, the missense variant p.R4810K (NM_001256071.3: c.14429G ​> ​A) is strongly associated with MMD (Guey et al., 2015). However, this association has not been replicated in other populations, as the p.R4810K variant is only present in Asian populations (https://gnomad.broadinstitute.org/variant/17-78358945-G-A) (Uchino et al., 2005; Ma et al., 2013; Liu et al., 2010, 2013a; Cecchi et al., 2014; Wang et al., 2013). Other rare *RNF213* missense variants have been identified in Caucasian MMD populations, suggesting that variants in the *RNF213* gene might confer some susceptibility, also in non-Asian patients (Guey et al., 2017a; Kobayashi et al., 2016; Zanoni et al., 2023). However, no single predominant susceptibility variant has been identified so far. Attempts have been made to identify variants responsible for the major effects in familial MMD, but findings have not been replicable (Wang et al., 2020). A genome wide association study (GWAS) was unsuccessful in identifying a predominant susceptibility variant for Caucasian MMD in contrast to the strong association of the variant p.R4810K in the *RNF213* gene in East-Asian populations (Liu et al., 2013a).

Based on the vast heterogeneity of reported findings in MMD patients of other than East-Asian origin, the aim of our pilot study was to establish an analysis strategy and genetically characterize MMD by WES in patients of Northern-European origin.

## 2 Methods

### 2.1 Ethics

The study was approved by the Regional Ethics Committee of South Eastern Norway (No. 2018/1377) and written informed consent was obtained from all study participants regarding publication of their data.

### 2.2 Patient recruitment

**Fig. 1:**
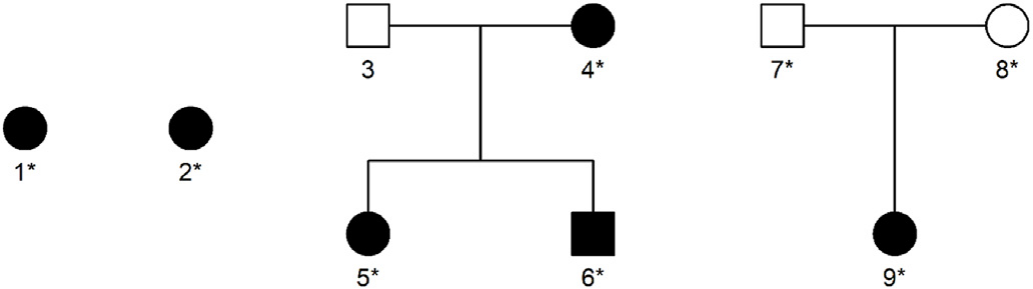
Overview of patients and families included in this study. Squares ​= ​men; circles ​= ​women. Filled symbols indicate MMD. Starred individuals (∗) were subjected to exome sequencing.

In this pilot study, adult patients of Northern-European origin, treated surgically for MMD at Oslo University Hospital (OUH) in the recruitment period (October 2018 to January 2019) were consecutively included. In familial cases, affected members were also asked to participate, and in one of the sporadic cases, healthy parents were included. Blood samples were drawn from all six patients and the healthy parents of one sporadic patient. The family structures of the collected patients are illustrated in Fig. 1.

All patients treated at OUH were thoroughly assessed at baseline, including medical history, family history, lifestyle factors, neurological examination, neuropsychological testing, laboratory tests and imaging. Symptomatic patients with hypoperfusion on MRI were offered surgical treatment with a combined direct and indirect revascularization. Diagnosis of MMD was based on international guidelines (Guidelines for diagnosis and treatment, 2012).

### 2.3 Whole exome sequencing

Whole exome sequencing (WES) was performed in all six patients and in two unaffected parents. Exome capture was done using the SureSelectXT V5 kit (Agilent, Böblingen, Germany), following the supplier's protocol for 3 ​μg DNA samples (Technologies, 2017). Paired end sequencing with read lengths of 150 bp was performed with Illumina HiSeq3000 technology (Illumina, San Diego, CA, USA).

### 2.4 Bioinformatic analysis and variant filtering

The sequences were aligned to the reference human genome (hg19) with bwa mem (v0.7.12) (Li and Durbin, 2010). The alignments were refined by the Genome Analysis Toolkit (GATK, v3.3) and PCR duplicates were marked by Picard (v1.124) (McKenna et al., 2010; Van der Auwera et al., 2013). Variant calling was performed with the Haplotype Caller feature of GATK (v3.3), applying joint calling for related individuals. The variants were annotated with ANNOVAR (v2017Jul16) (Wang et al., 2010) while filtering and downstream analysis was done in FILTUS (Vigeland et al., 2016).

### 2.5 Analysis strategies for the identification of genetic variants associated with MMD

**Fig. 2:**
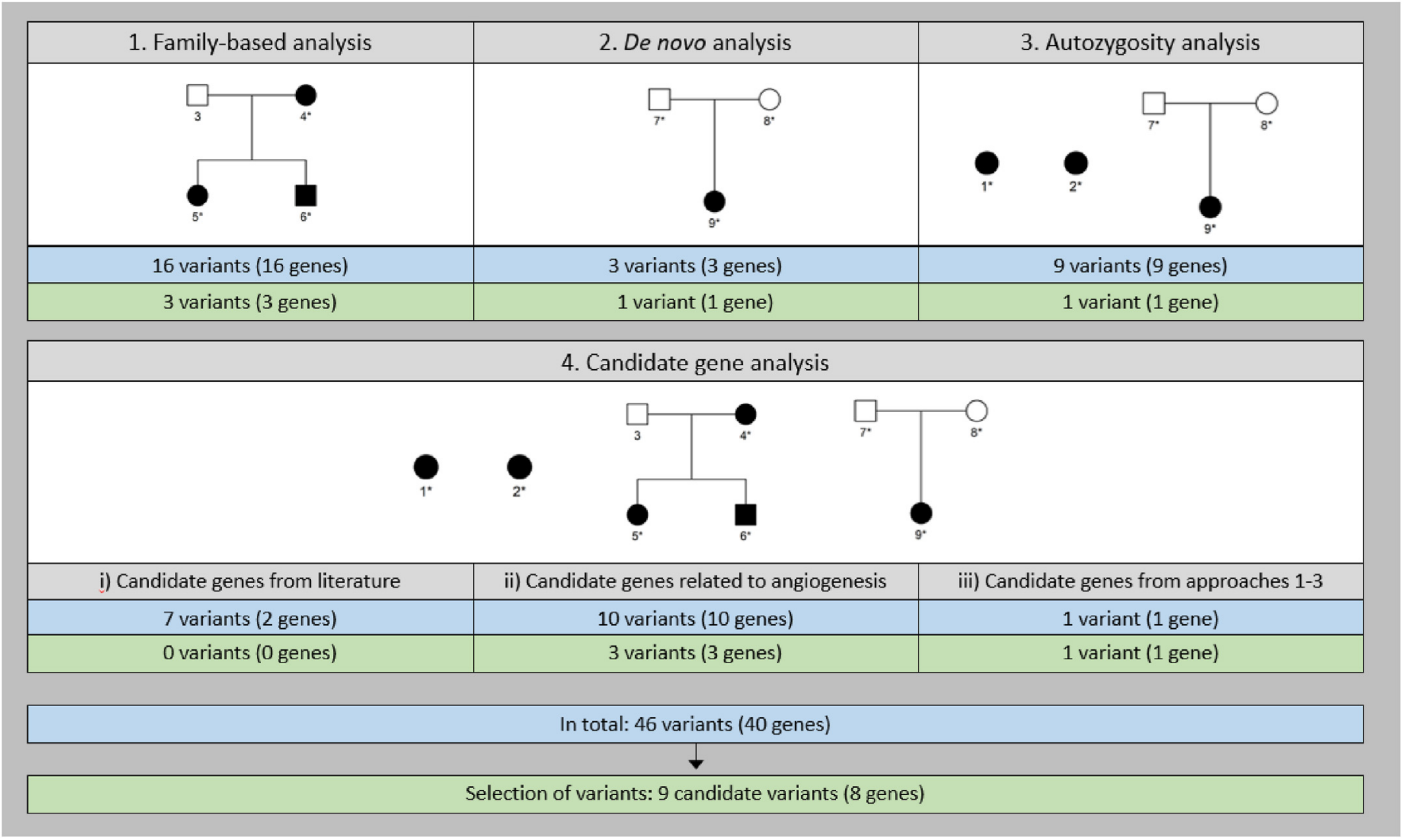
Overview of analysis approaches and variant filtering. Blue boxes are the results from the filtering with the cut-offs given in Table 1, whereas the green boxes are the results from discretionary selection of variant after review of literature and results from in silico prediction tools. (For interpretation of the references to colour in this figure legend, the reader is referred to the Web version of this article.)

Our analysis strategy was tailored to each patient, according to family structure and sample availability. The four different approaches are summarized in Table 1 and illustrated in Fig. 2. Approach 1: a family-based analysis was applied for the three patients in the same family, applying a dominant model, i.e., identification of all joint heterozygous variants. Approach 2: *de novo* mutation analysis was performed for the patient with available parental samples. Approach 3: autozygous regions were identified in all three non-familial cases, using the AutEx algorithm of the FILTUS program. Though consanguinity was not suspected in any of the patients, this analysis was included to identify potential cryptic consanguinity, i.e., homozygous regions resulting from unknown distant parental relationships. Approach 4: Analysis of candidate genes was performed in all patients. We applied three candidate gene lists, based on three sources: i) 27 candidate genes previously reported in MMD (see Supplementary Table 2); ii) 147 genes known to be involved in angiogenesis, i.e., a selection based on gene lists made for QIAGEN array (complete list in Supplementary Table 3); and iii) the genes in which variants were identified by analysis approach 1, 2 or 3 were re-analysed in all patients using the more liberal filters for approach 4 (Table 1).

**Table 1:** Filtering strategies for identification of genetic variants associated with MMD.

Analysis	Patients	Region	Depth	Allele frequency	Comments
***1. Family-based, dominant model***	4, 5, 6	Exon/splice site	>5	<0.0001	Strict frequency filter justified by dominant disease model
***2. De novo***	9	Exon/splice site	>9	<0.01	Higher depth to avoid false positives. Mutation rate set to 10^−8^
***3. Autozygosity***	1, 2, 9	Exon/splice site	>5	<0.015	Relaxed frequency filter justified by autozygous disease model
***4. Candidate genes***	All patients	Exon/splice site	>5	<0.01	Selected genes from the literature and analyses 1-3

After filtering and analysing in FILTUS, a discretionary review of the candidate variants was performed based on literature review of the genes combined with predicted impact on protein function using several prediction tools including GERP++ (Davydov et al., 2010), SIFT (Kumar et al., 2009), PolyPhen (v2) (Adzhubei et al., 2013), LRT (Chun and Fay, 2009), MutationTaster (Schwarz et al., 2010), MutationAssessor (Reva et al., 2011), FATHMM-XF (Rogers et al., 2018), PROVEAN (Choi and Chan, 2015) and the CADD score (Rentzsch et al., 2019; Kircher et al., 2014).

## 3 Results

### 3.1 Study cohort

Six adult patients of Northern-European origin, diagnosed with MMD were included. The study cohort consisted of three unrelated sporadic cases and one family with MMD. The family included three confirmed MMD family members: one mother and two adult children (Fig. 1). Blood samples were obtained from all six patients and from the two unaffected parents, i.e., 8 blood samples in total.

Five out of six patients were female, and mean age was 33 years (range 23–56). All patients had experienced at least one ischemic stroke, whereas the three familial patients had multiple TIAs or strokes. Headaches and fatigue were the most common chronic symptoms, reported in four of the patients. Five of the patients received both medical and surgical treatment with revascularization, while one patient had refused treatment.

### 3.2 Genetic analysis

The results of genetic analyses and filtering are summarized in Fig. 2. After WES, analysis approaches 1–3 resulted in 28 genes of interest: 16 variants in 16 genes of interest from approach 1 (family-based); three variants in three genes from approach 2 (*de novo* analysis) and 9 variants in 9 genes resided in an autozygous region in individual 1 (approach 3, autozygous regions). Next, analysis approach 4, the candidate gene analysis, yielded variants in 13 genes: 7 variants in 2 genes (gene list based on literature); 10 variants in 10 genes (list of candidate genes involved in angiogenesis), and finally only one variant was identified in candidate genes included from analyses 1–3. In total, we identified 46 variants in 40 genes of interest. Of note, one gene (*NR4A3*) is listed in both approach 1 and in approach 4 iii). A discretionary review of these variants narrowed the list to nine variants in eight genes (Table 2). Of these eight genes, five encode proteins involved in NO metabolism: *NOS3*, *NR4A3, ITGAV, GRB7* and *AGXT2*. A list of the 32 genes not selected by the last discretionary selection can be found in Supplementary Table 4.

**Table 2:** Genetic variants identified in the study cohort.

Gene	Variant	Frequency (gnomAD, popmax)	Identification method	CADD score	Patients
***NR4A3***	NM_006981: c.C872T; p.Thr291Met	0.00006	Family-based analysis	24.8	4, 5, 6
***NR4A3***	NM_006981: c.C1578G; p.Ser526Arg	0.00014	Candidate gene approach (Re-analysis of genes identified in approaches 1–3)	16.01	2
***PDZRN3***	NM_001303139: c.C1362G; p.Ser454Arg	Not previously observed	Family-based analysis	25.6	4, 5, 6
***TNFAIP2***	NM_006291: c.C1240G; p.Gln414Glu	Not previously observed	Family-based analysis	12.09	4, 5, 6
***AGXT2***	NM_001306173: c.C703T; p.Pro235Ser	Not previously observed	*De novo* analysis	13.9	9
***NOS3***	NM_001160109: c.G1684A; p.Glu562Lys	0.00003	Candidate gene approach (angiogenesis gene)	33	1
***ITGAV***	NM_001145000: c.A1313T; p.Asn438Ile	Not previously observed	Candidate gene approach (angiogenesis gene)	26.8	1
***FGR3***	NM_000142: c.A1136G; p.Tyr379Cys	Not previously observed	Candidate gene approach (angiogenesis gene)	25.3	2
***GBR7***	NM_001030002: c.G869A; p.Arg290His	0.00089	Autozygous region	32	1

## 4 Discussion

In this study, whole exome sequencing in six MMD patients and two healthy parents of Northern-European origin was performed, including different analysis strategies (Table 1). We identified variants in eight candidate genes (Table 2). Remarkably, five of these genes are involved in NO regulation, where one gene, the *AGXT2* gene, was identified as a *de novo* variant in one of the sporadic cases. *AGXT2* has not been previously described as a gene that confers increased susceptibility for MMD, and the identification of this gene through an unbiased analysis (*de novo* analysis) thus corroborates the hypothesis of NO pathway involvement in MMD pathophysiology.

Several variants of the *RNF213* gene are strongly associated with MMD in East-Asia (Liu et al., 2011; Kamada et al., 2011; Ma et al., 2013) and rare missense variants have been reported in non-Asian MMD (Zanoni et al., 2023; Guey et al., 2017b). However, we did not identify the p.R4810K variant of the *RNF213* gene in any of the patients, nor any other potential risk variants in this gene. This finding is in line with previous reports on non-Asian MMD, not confirming predominant susceptibility variants (Cecchi et al., 2014; Guey et al., 2017a; Liu et al., 2013b).

**Fig. 3:**
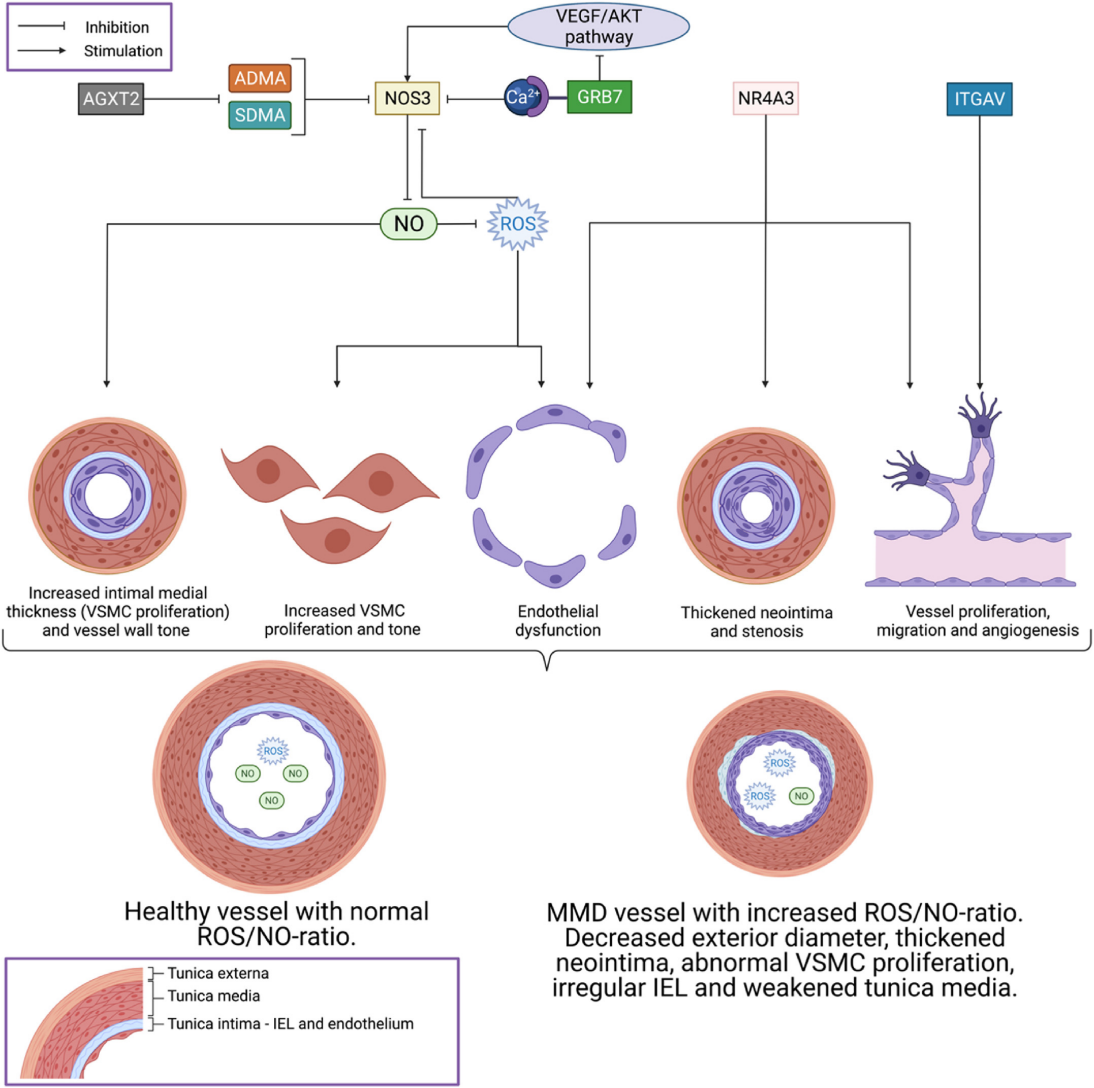
Suggested involvement of *NR4A3, AGXT2, NOS3, ITGAV* and *GRB7* in the development of vessel wall pathology and angiogenesis in moyamoya disease. (Abbreviations: VSMC ​= ​vascular smooth muscle cell; IEL ​= ​internal elastic lamina; MMD ​= ​moyamoya disease; NO = Nitric oxide; ROS ​= ​reactive oxygen species.) Created with BioRender.com.

The eight identified candidate genes were selected based on rare frequencies in the population, and prediction of pathogenic effects on protein function, including the CADD score (Rentzsch et al., 2019). Five of the identified candidate genes may influence endothelial NO regulation (Fig. 3). NO-dependent pathways are crucial for healthy angiogenesis, and NO plays a protective role as an endogenous vasodilator by regulating normal blood vessel tone and diameter (Rochette et al., 2013). Reduced NO bioavailability in the endothelium results in decreased vasodilation by reduced vascular smooth muscle cell (VSMC) relaxation (Davignon and Ganz, 2004). Loss of NO triggers VSMC proliferation and migration, leading to vessel stenosis (Northcott et al., 2017).

Endothelial-derived NO is crucial for angiogenesis, suggesting that variants in genes encoding proteins involved in the regulation of NO levels are plausible candidates for MMD pathology. Moreover, some evidence of dysfunctional NO regulation in MMD patients exists: Biallelic variants in the gene *GUCY1A3* encoding a NO receptor have been shown to cause MMD associated with achalasia in European patients (Herve et al., 2014; Wallace et al., 2016), and recently, biallelic variants in NOS3 were identified in two consanguineous probands with moyamoya angiopathy (Guey et al., 2023). NO metabolites are further elevated in the cerebrospinal fluid of patients with MMD (Noda et al., 2000).

NO is produced by NO-synthases (NOS) (Rochette et al., 2013), and NOS3 produces NO in the endothelium. Variants in the *NOS3* gene in MMD patients may thus represent a possible link to disease phenotype. It has been shown that imbalanced *NOS3* synthesis leads to endothelial dysfunction by increased production of reactive oxygen species (ROS) (Rochette et al., 2013). Increased ROS levels then lead to oxidative stress in cells (Rochette et al., 2013; Alonso et al., 2018). Moreover, ROS can oxidize a cofactor of *NOS3*, which again prevents the formation of the *NOS3* dimer (Rochette et al., 2013). *NOS3* then contributes to a further increased ROS production, thus creating a vicious circle (Rochette et al., 2013).

NOS3 is also activated by an elevation in intracellular calcium (Andrew and Mayer, 1999). Through this signalling pathway, mutations in *Grb7* may cause endothelial dysfunction through reduced angiogenetic activity both through changes in intracellular calcium signalling and the VEGF/Akt (PKB)/NO pathway (Andrew and Mayer, 1999; Sahlberg et al., 2013).

The naturally occurring amino acids ADMA and SDMA, are endogenous inhibitors of NOS and can inhibit NOS competitively (Rees et al., 1990; Faraci et al., 1995) or partly by reducing substrate availability (Boger et al., 2000; Closs et al., 1997). *AGXT2* metabolizes both ADMA and SDMA (MacAllister et al., 1996; Seppala et al., 2016), and lower *AGXT2* activity is associated with higher risk for cardiovascular disease and increased carotid intimal medial thickness (Yoshino et al., 2014). Reduced *AGXT2* activity may lower the NO-producing activity of *NOS3*, through a higher concentration of ADMA and SDMA. Elevated serum levels of ADMA and SDMA have been shown to reduce NO bioavailability leading to endothelial dysfunction (Sitia et al., 2010; Tain et al., 2010; Vallance et al., 1992; Boger et al., 2009; Feliers et al., 2015) and intimal hyperplasia (Masuda et al., 1999), and to increase superoxide production in endothelial cells (Antoniades et al., 2009; Wilcox, 2012).

The transcription factor NR4A3 belongs to the NR4A subfamily of the nuclear hormone receptor superfamily, and is implicated in angiogenesis, induces endothelial cell growth (Hirano et al., 2019; Mohan et al., 2012; Wenzl et al., 2015; Crean and Murphy, 2021; Rius et al., 2004, 2006; Martinez-Gonzalez et al., 2003; Arkenbout et al, 2002, Arkenbout et al, 2003) and is modulated through several pathways, including PI3K/Akt (PKB). Up-regulation by NR4A3 causes endothelial dysfunction by promoting vessel proliferation and migration (Wang et al., 2021), as well as thickened neointima and increasing stenosis after vascular damage (Rodríguez-Calvo et al., 2013).

*ITGAV* encodes a αVβ3 heterodimer subunit. αVβ3 has been implicated in regulation of endothelial cell adhesion, migration and survival and may play a key role in angiogenesis (Avraamides et al., 2008; Mahabeleshwar et al., 2006; Ruegg and Mariotti, 2003). The αVβ3 heterodimer is highly expressed in proliferative endothelial cells and is up-regulated in tumorigenic blood vessels (Liao et al., 2017; Felding-Habermann and Cheresh, 1993; Plow et al., 2014). Blocking αVβ3 function reduces angiogenesis and capillary network size (Friedlander et al., 1995; Mas-Moruno et al., 2010; Fukushima et al., 2005). VEGF up-regulates the ITGAV/ITGB3 subunit (Witmer et al., 2004). Recently, genes regulating chromatin remodelling have been shown to be associated with moyamoya angiopathy (Pinard et al., 2020). NO seems to play a key role in chromatin folding in human vascular endothelial cells (Illi et al., 2008). This may suggest that several possible pathophysiological mechanisms underlying MMD seem to converge through the pathway of NO dysregulation.

The identified gene variants in our study have not been associated with other neurovascular disorders, however, several have an impact on NO pathways. Thus, NO pathways may well represent a key mechanism in MMD pathology. Our findings are in line with a complex mode of inheritance and underpin previous findings where one distinct predominant susceptibility gene is absent in Northern-European MMD patients.

Our study has several limitations. The main limitation is the small number of patients with different family structures, posing a particular challenge to our study with regards to the choice of analytical approach. Given our small and heterogeneous cohort, our results must be considered hypothesis generating and warranting confirmation from larger studies. It also needs to be considered that the selection of genes associated with angiogenesis in our candidate gene list may bias our results towards a false positive finding in regards to NO regulation pathways involved in MMD pathogenesis. Finally, a restriction of WES is the exclusive identification of variants in the *coding* regions of the genome. Variations in non-coding regions as well as large structural changes, such as large indels, duplications of genes and CNV remain undetected.

The candidate genes identified in this study add to the growing list of candidate genes in MMD. Moreover, the recent finding of biallelic *NOS3* variants in familial moyamoya angiopathy (Guey et al., 2023) underlines the importance of the NO pathways and warrants future functional exploration of the consequences of the variants identified. In addition to the genes identified in the current report, other genes associated with NO pathways and vascular regulation in MMD would also be valuable candidates for future functional studies. For instance, homozygous variants in the gene *GUCY1A3* increased the risk for MMD in a small sub-set of European patients with achalasia (Wallace et al., 2016). *GUCY1A3* encodes a subunit of the soluble guanylyl cyclase (SGC) complex which acts as an important receptor for NO (Kessler et al., 2017). Inactivation of genes encoding subunits of the SGC complex has been shown to reduce angiogenesis *in vitro* (Saino et al., 2004). Recently, *DIAPH1* variants have been shown to be associated with sporadic MMD in Non-East Asian patients in a genetic association study using WES (Kundishora et al., 2021). The results from the latter study suggest *DIAPH1* as a novel risk gene for MMD by association with impaired vascular cell actin remodelling in MMD pathogenesis (Kundishora et al., 2021).

## 5 Conclusion

No shared genetic risk variant or gene was identified in our patients of Northern-European origin, and none had the East-Asian variant in the ring finger protein *RNF213.* Identification of a *de novo* variant in the *AGXT2* gene supports the hypothesis of NO pathway dysregulation as a key contributor and plausible risk factor in MMD pathophysiology. This is further supported by the identification of five other rare gene variants in *NR4A3, ITGAV, GRB7* and *NOS3,* all involved in NO metabolism. The role of NO metabolism in MMD should be further assessed in functional studies and confirmed in larger patient cohorts.

## Compliance with ethical standards

No funding was received for this research.

All procedures performed in studies involving human participants were in accordance with the ethical standards of the National Hospital, Oslo University Hospital, the Regional Ethics Committee of South Eastern Norway and with the 1964 Helsinki declaration and its later amendments.

## Declaration of competing interest

MW: research grants from the 10.13039/501100006095South-Eastern Norway Regional Health Authority (grant number 2014060), ownership of stock Biontech/Pfizer, speaker honoraria from the 10.13039/100010745Norwegian Medical Association.

The other authors declare no conflict of interest.
