# Supplementary material for: Whole-exome sequencing in moyamoya patients of Northern-European origin identifies gene variants involved in Nitric Oxide metabolism: A pilot study
Source: Brain Spine. 2023 Apr 22;3:101745. doi: 10.1016/j.bas.2023.101745 (PMC10293314; doi:10.1016/j.bas.2023.101745)
Supplement: Multimedia component 1 [file mmc1.docx]

**Supplementary table 1.** Previously identified candidate genes in Caucasian MMD patients and proposed gene function

| **Candidate gene** | **Role in MMD** | **Gene function** |
| --- | --- | --- |
| ***RNF213*[1, 2]** | One variant is associated with Asian MMD, other variants have been found in Caucasian MMD. | Inhibition of angiogenesis by inhibition of the Wnt signalling pathway and promotion of vessel regression. |
| ***GUCY1A3*[3, 4]** | Homozygous variants lead to MMS associated with achalasia. Some variants lead to MMD and hypertension. | Sub-unit in the main receptor of NO. Regulates relaxation of smooth muscle cells and platelet function. |
| ***ACTA2*[5, 6]** | Some variants present in MMD-patients, while one study found none of the described variants. | Contraction of vascular smooth muscle cells important for arterial structural integrity. |
| ***PDGFRB*[7]** | One SNP in the promoter region associated with MMD. | Signalling in cells mesenchymal origin. Normal development of the cardiovascular system. |
| ***TGFB1*[7]** | One SNP in exon 1 associated with MMD. | Angiogenesis, muscle development and inflammatory processes. |
| ***ZXDC*[8]** | One variant present in 10 of 68 Caucasian MMD-patients, with a frequency of 4% in Caucasian populations. | Promotes (with *CIITA*) translocation of MHC class 1- and MHC class 2-genes. |
| ***OBSCN*[8]** | One of the most enriched genes in a Caucasian population shown by collapsing variant methodology. | May be involved in synthesis of myofibrils in muscle cells. One mutation is associated with hypertrophic cardiomyopathy. |
| ***CBL*[9]** | *De novo*-variant identified in one Caucasian trio and one non-Caucasian trio with MMA. | Negative regulator for several signal pathways (PDGFRB amongst others). Signal transduction in hematopoietic cells. |

**Supplementary table 2.** Candidate genes from the literature

| **Candidate genes in Caucasian MMD** | **Candidate genes in Asian MMD** |
| --- | --- |
| *RNF213* [1, 2]  *GUCY1A3* [3, 4]  *ACTA2* [5, 6]  *PDGFRB, TGFB1* [7]  *NF1* [10]  *OBSCN, ZXDC* [8]  *CBL* [9] | *RNF213* [11, 12]  *CARD14, CTAGE1, FADS1, FADS2, HDAC9, HORMAD2, JAZF1, LRP1, MTHFR, PLOD1, SBF2, SPTLC3, TCN2, TSC22D2* [13]  *TIEG/KLF10* [14]  *RPTOR* [13, 15]  *TIMP2* [16] |

**Supplementary table 3.** Candidate genes involved in angiogenesis by Qiagen (list available from Qiagen upon request)

| *A2M*  *ADORA2B*  *AGT*  *AGTR2*  *ANG*  *ANGPT1*  *ANGPT2*  *BMX*  *BRAF*  *CAV1*  *CAV2*  *CD36*  *CD151*  *CDH5*  *CHGA*  *COL8A1*  *COL8A2*  *CTGF*  *CTSB*  *ECGF1*  *EDG1*  *EDG3*  *EDN1*  *EFNB2*  *EGF*  *EGFR*  *EGR1*  *ENG*  *EPAS1*  *EPHB4*  *EPO*  *ERBB2*  *ETS1*  *FGF1*  *FGF2*  *FGF4*  *FGF6* | *FGF7*  *FGFR1*  *FGFR3*  *FGFR4*  *FIGF*  *FLT1*  *FLT4*  *FN1*  *FNTA*  *FNTB*  *GATA2*  *GPR30*  *HGF*  *HIF1A*  *TNC*  *ID1*  *ID3*  *IL8*  *ITGAV*  *ITGB3*  *KDR*  *MCAM*  *DNAJB9*  *MDK*  *MMP2*  *MMP3*  *MMP9*  *NOS3*  *NRAS*  *SERPINE1*  *SERPINB2*  *PDGFB*  *PDGFRA*  *PDGFRB*  *PECAM1*  *SERPINF1*  *PF4* | *PGF*  *PGGT1B*  *SERPINB5*  *PLAU*  *PLAUR*  *MAPK7*  *PTAFR*  *PTGS1*  *PTGS2*  *PTN*  *SELE*  *SELP*  *SPARC*  *SPP1*  *TEK*  *TGFBR1*  *TGFBR2*  *TGFBR3*  *THBS1*  *THBS2*  *THBS3*  *THBS4*  *TIE1*  *TIMP1*  *TIMP2*  *TIMP3*  *TIMP4*  *TNFAIP1*  *TNNI3*  *TNNT2*  *VCAM1*  *VEGFA*  *VEGFB*  *VEGFC*  *SPARCL1*  *SCARF1*  *PPAP2B* | *EDG6*  *EDF1*  *NRP2*  *NRP1*  *ANGPTL1*  *SCYE1*  *EDG5*  *LIPG*  *ADAMTS1*  *ARHGEF17*  *TNFSF15*  *EDIL3*  *ESM1*  *ADAMTS8*  *ANGPTL2*  *RASD2*  *GPR124*  *ANGPTL3*  *REM1*  *ANGPTL4*  *ANGPT4*  *EDG8*  *ZNF444*  *CD248*  *PLXDC1*  *ZNF71*  *JAM2*  *TNS3*  *ANGPTL6*  *ANTXR1*  *PROK1*  *PLXDC2*  *ESAM*  *SCARF2*  *DCBLD2*  *ANGPTL5*  *OR5AT1* |
| --- | --- | --- | --- |

**Supplementary table 4.** List of the 32 genes not selected after the last discretionary selection.

| **Gene name** | **Gene description** | **# of variants** |
| --- | --- | --- |
| **1. Family-based analysis** | | |
| *GALNT16* | UDP-N-ACETYL-ALPHA-D-GALACTOSAMINE:POLYPEPTIDE N-ACETYLGALACTOSAMINYLTRANSFERASE 16 | 1 |
| *RFTN1* | RAFTLIN | 1 |
| *RTN1* | RETICULON 1 (NEUROENDOCRINE-SPECIFIC PROTEIN) | 1 |
| *PHLDB1* | PLECKSTRIN HOMOLOGY-LIKE DOMAIN, FAMILY B, MEMBER 1 | 1 |
| *NR4A3* | CHONDROSARCOMA, EXTRASKELETAL MYXOID, FUSED TO EWS IN | 1 |
| *CNGB1* | CYCLIC NUCLEOTIDE GATED CHANNEL, BETA 1 | 1 |
| *SRCAP* | SNF2-RELATED CBP ACTIVATOR PROTEIN | 1 |
| *MAP4K5* | MITOGEN-ACTIVATED PROTEIN KINASE-5 | 1 |
| *COL22A1* | COLLAGEN, TYPE XXII, ALPHA-1 POLYPEPTIDE | 1 |
| *AURKB* | SERINE/THREONINE PROTEIN KINASE 12 | 1 |
| *SASS6* | SAS6, C. ELEGANS, HOMOLOG OF | 1 |
| *CTRL* | CHYMOTRYPSIN-LIKE PROTEASE | 1 |
| *GPR88* | G-PROTEIN COUPLED RECEPTOR 88 | 1 |
| **2. *De novo* analysis** | | |
| *KLHL24* | KELCH-LIKE 24 | 1 |
| *DMC1* | DISRUPTED MEIOTIC CDNA 1, YEAST, HOMOLOG OF | 1 |
| **3. Autozygosity analysis** | | |
| *KRT33B* | KERATIN 33B | 1 |
| *PLIN5* | PERILIPIN 5 | 1 |
| *TLE2* | TRANSDUCIN-LIKE ENHANCER OF SPLIT 2 (HOMOLOG OF DROSOPHILA E(SPL)) | 1 |
| *ANKRD40* | ANKYRIN REPEAT DOMAIN 40 | 1 |
| *ECEL1* | ENDOTHELIN-CONVERTING ENZYME-LIKE 1 | 1 |
| *IFI35* | INTERFERON-INDUCED PROTEIN-35 | 1 |
| *SYNRG* | ADAPTOR-RELATED PROTEIN COMPLEX 1 GAMMA SUBUNIT-BINDING PROTEIN 1 | 1 |
| *ARRDC5* | ARRESTIN DOMAIN CONTAINING 5 | 1 |
| **4. i) Candidate genes from literature** | | |
| *OBSCN* | OBSCURIN | 5 |
| *LRP1* | LOW DENSITY LIPOPROTEIN-RELATED PROTEIN-1 (ALPHA-2-MACROGLOBULIN RECEPTOR) | 2 |
| **4. ii) Candidate genes related to angiogenesis** | | |
| *FLT4* | FMS-RELATED TYROSINE KINASE-4 (VASCULAR ENDOTHELIAL GROWTH FACTOR RECEPTOR 3) | 1 |
| *FN1* | FIBRONECTIN-1 | 1 |
| *SELP* | SELECTIN P (GRANULOCYTE MEMBRANE PROTEIN, 140KD; ANTIGEN CD62) | 1 |
| *EGF* | EPIDERMAL GROWTH FACTOR (UROGASTRONE) | 1 |
| *ERBB2* | AVIAN ERYTHROBLASTIC LEUKEMIA VIRAL (V-ERB-B2) ONCOGENE HOMOLOG 2 (NEURO/GLIOBLASTOMA DERIVED ONCOGENE HOMOLOG) | 1 |
| *EPAS1* | ENDOTHELIAL PAS DOMAIN PROTEIN 1 | 1 |
| *CD248* | CD248 ANTIGEN | 1 |
| **4. iii) Candidate genes from approaches 1-3** | | |
|  |  | 0 |

**Citations (Supplementary material)**

1. Kobayashi, H., et al., *RNF213 Rare Variants in Slovakian and Czech Moyamoya Disease Patients.* PLoS One, 2016. **11**(10): p. e0164759.

2. Guey, S., et al., *Rare RNF213 variants in the C-terminal region encompassing the RING-finger domain are associated with moyamoya angiopathy in Caucasians.* Eur J Hum Genet, 2017. **25**(8): p. 995-1003.

3. Herve, D., et al., *Loss of alpha1beta1 soluble guanylate cyclase, the major nitric oxide receptor, leads to moyamoya and achalasia.* Am J Hum Genet, 2014. **94**(3): p. 385-94.

4. Wallace, S., et al., *Disrupted nitric oxide signaling due to GUCY1A3 mutations increases risk for moyamoya disease, achalasia and hypertension.* Clin Genet, 2016. **90**(4): p. 351-60.

5. Guo, D.C., et al., *Mutations in smooth muscle alpha-actin (ACTA2) cause coronary artery disease, stroke, and Moyamoya disease, along with thoracic aortic disease.* Am J Hum Genet, 2009. **84**(5): p. 617-27.

6. Roder, C., et al., *Analysis of ACTA2 in European Moyamoya disease patients.* Eur J Paediatr Neurol, 2011. **15**(2): p. 117-22.

7. Roder, C., et al., *Polymorphisms in TGFB1 and PDGFRB are associated with Moyamoya disease in European patients.* Acta Neurochir (Wien), 2010. **152**(12): p. 2153-60.

8. Shoemaker, L.D., et al., *Disease Variant Landscape of a Large Multiethnic Population of Moyamoya Patients by Exome Sequencing.* G3 (Bethesda), 2015. **6**(1): p. 41-9.

9. Guey, S., et al., *De novo mutations in CBL causing early-onset paediatric moyamoya angiopathy.* J Med Genet, 2017. **54**(8): p. 550-557.

10. Serafini, N.B., et al., *Moyamoya syndrome associated with neurofibromatosis type 1 in a pediatric patient.* An Bras Dermatol, 2017. **92**(6): p. 870-873.

11. Kamada, F., et al., *A genome-wide association study identifies RNF213 as the first Moyamoya disease gene.* J Hum Genet, 2011. **56**(1): p. 34-40.

12. Liu, W., et al., *Identification of RNF213 as a susceptibility gene for moyamoya disease and its possible role in vascular development.* PLoS One, 2011. **6**(7): p. e22542.

13. Duan, L., et al., *Novel Susceptibility Loci for Moyamoya Disease Revealed by a Genome-Wide Association Study.* Stroke, 2018. **49**(1): p. 11-18.

14. Sakurai, K., et al., *A novel susceptibility locus for moyamoya disease on chromosome 8q23.* J Hum Genet, 2004. **49**(5): p. 278-81.

15. Liu, W., et al., *A rare Asian founder polymorphism of Raptor may explain the high prevalence of Moyamoya disease among East Asians and its low prevalence among Caucasians.* Environ Health Prev Med, 2010. **15**(2): p. 94-104.

16. Kang, H.S., et al., *Single nucleotide polymorphisms of tissue inhibitor of metalloproteinase genes in familial moyamoya disease.* Neurosurgery, 2006. **58**(6): p. 1074-80; discussion 1074-80.
